# Linking Self-Control to Voluntary Behaviors at Workplace: The Mediating Role of Job Satisfaction

**DOI:** 10.3389/fpsyg.2021.530297

**Published:** 2021-03-23

**Authors:** Yu-Jie Wang, Kui-Yun Chen, Kai Dou, Yao-Zhong Liu

**Affiliations:** ^1^Mental Health Education and Counseling Center, Guangdong Industry Polytechnic, Guangzhou, China; ^2^School of Health in Social Science, The University of Edinburgh, Lothian, United Kingdom; ^3^Department of Psychology, School of Education, Guangzhou University, Guangzhou, China; ^4^School of Economics and Management, Guangzhou Huashang College, Guangzhou, China

**Keywords:** self-control, job satisfaction, voluntary work behavior, OCB, CWB, employee

## Abstract

Voluntary work behavior (VWB) refers to spontaneous workplace behaviors that extend beyond role norms, including extra-role behaviors that benefit the organization (i. e., organizational citizenship behavior, OCB) and negative behaviors that may harm the organization (i.e., counterproductive work behavior, CWB). This study examined the relationship between self-control and VWB and the mediating role of job satisfaction. A total of 1,101 full-time employees from China completed a battery of self-report measures online. The results show that self-control positively predicts employees' OCB and negatively predicts employees' CWB. Moreover, job satisfaction significantly mediates the relationship between self-control and OCB/CWB. The results confirm that employees with high self-control are more public-spirited, which previous studies have described as being “highly committed” (high OCB) or “less harmful” (low CWB). This finding closely relates to the observation that employees with high self-control tend to have more satisfying work outcomes or higher workplace status than those with low self-control.

## Introduction

Voluntary work behavior (VWB) refers to spontaneous behaviors that extend beyond specific role requirements, including desirable (i.e., organizational citizenship behavior, OCB) and undesirable behaviors (i.e., counterproductive work behavior, CWB) (Fay and Sonnentag, 2010; Mekpor and Dartey-Baah, 2017). OCB is employees' behavior that extends beyond usual job duties and expectations and that can promote organizational effectiveness (Organ et al., 2010; Miao et al., 2020). It is associated with a range of positive organizational-level outcomes (e.g., productivity, efficiency, reduced costs, and customer satisfaction) (Podsakoff et al., 2009). CWB refers to behavior that is intended to have a negative effect on organizations and their members (Fox et al., 2001). It can take many different forms, such as theft, fraud, absenteeism, physical aggression, and substance use (Marcus and Schuler, 2004), which can cause a large share of organizational losses (Braun et al., 2016).

Over the last decade, growing empirical research has highlighted other environmental factors that may affect VWB, such as leadership style and organizational justice (Wu et al., 2016; Newman et al., 2017), and that are typically observed at the organizational level. However, research on the impact of personal factors on VWB is very limited (Guan et al., 2019). One personal trait, self-control, has been a prominent concept in different areas of research in psychology because of its various beneficial effects for human functioning (de Ridder et al., 2012). The famous Chinese idiom “self-controlling and public-spirited” links self-control ability to commitment to the public interest. This context leads us to ask whether self-control ability will lead to more behaviors that will benefit the organization. Correspondingly, will self-control ability be related to less CWB? In the current study, we aim to explore the relationship between self-control and OCB/CWB and the possible mediating role of job satisfaction.

## Literature Review and Hypothesis Development

### Self-Control and OCB/CWB

Self-control is the capacity to override, modify, or suppress desirable behavior when pursuing long-term goals (de Ridder et al., 2012; Situ et al., 2016). Individuals who have higher levels of self-control tend to experience more positive outcomes (such as better interpersonal relationships and better emotional responses) and fewer negative outcomes (such as less binge eating, alcohol abuse, crime, and juvenile delinquency) (Tangney et al., 2004; Walters, 2016; Wolfe et al., 2016). OCB refers to behavior in which employees voluntarily sacrifice their short-term interests for the organization's long-term interests (Joireman et al., 2006). When choosing to demonstrate OCB, such as helping colleagues and saving organizational resources, employees may have to resist instinctive impulses toward selfishness. To override their automatic selfish impulse, individuals need to utilize the ability of self-control (Dewall et al., 2008). Correspondingly, studies have shown that it is easier for people with higher levels of self-control to regulate negative emotions, manage conflict (Hofmann et al., 2014), control impulses and temptation (de Ridder et al., 2012), and achieve long-term goals (Frederick et al., 2003). OCB is related to the psychological process of self-control, which includes overcoming inner resistance, resisting distractions, and suppressing impulsiveness (Diestel and Schmidt, 2012; Rivkin et al., 2015). This view is supported by recent empirical studies suggesting that self-control is positively related to OCB (Joosten et al., 2015; Wang et al., 2017). As noted above, we hypothesize the following:

***Hypothesis 1a****: Self-control is positively related to OCB*.

When employees engage in CWB, such as purposely performing work incorrectly, being late, and stealing from the organization, they are potentially harming the interests of the organizations or its members (Marcus and Schuler, 2004; Shoss et al., 2016). What motivates individuals to display these harmful acts? Previous studies have suggested that CWB is a behavioral strain response to provocation at work (i.e., workplace stress, organizational constraints, and interpersonal conflict) because individuals anticipate that these behaviors will make them feel better emotionally (Fox et al., 2001; Spector and Fox, 2002). CWB is an attempt to cope with stress and negative events, which may relieve employees' negative emotions (Reynolds et al., 2015). To avoid displaying CWB, employees need to utilize self-control ability to minimize the undesirable impact of stress and negative emotions, suggesting that low self-control is linked to counterproductive work behavior (Marcus and Schuler, 2004; Hofmann et al., 2014). Given the reasons mentioned above, we assume that employees who have a higher level of self-control can regulate their personal emotions and therefore display less CWB. Thus, we hypothesize the following:

***Hypothesis 1b****: Self-control is negatively related to CWB*.

### Job Satisfaction as a Mediator

Although there may be various mechanisms whereby self-control relates to OCB and CWB, we would argue that one of the mechanisms is carried through the mediating role of employees' job satisfaction. Job satisfaction refers to the positive feelings about one's working conditions or outcomes that arise from one's work (Saari and Judge, 2004). Despite the large amount of literature suggesting the benefits of self-control in various aspects, such as work, academic performance, and interpersonal relationships (de Ridder et al., 2012), remarkably limited research concerned the possible links between self-control and subjective well-being (Hofmann et al., 2014). Hofmann et al. (2014) reported that people with higher levels of self-control are more likely to be satisfied with their lives because they are better able to regulate negative emotions, manage conflict, and balance their daily lives. This argument leads us to suggest that employees' self-control ability can also contribute to their job satisfaction as work takes up a large part of life. As a significant part of life for adults, work is an important factor that can influence people's perception about their lives and well-beings. However, to the best of our knowledge, little study has directly examined the relationship between self-control and job satisfaction. A preliminary study confirmed the positive relationship between self-control and job satisfaction in a Chinese sample working in different cities (Dou et al., 2016). Another study by Rothman and Coetzer (2002) also suggested individuals' personality traits, such as self-discipline, can influence one's job satisfaction through cognitive, affective, and behavioral process. The possible explanation could be that the ability of self-control may lead to better regulation of employees' daily lives, including negative emotions, stress, and interpersonal relationships, which might be associated with higher job satisfaction.

Well-established literature has demonstrated the positive relationship between job satisfaction and OCB. An important driver of OCB is the extent to which employees feel satisfied with their job (Bowling, 2009). For example, a recent study found that employees' job satisfaction can enhance their willingness to perform positive work activities (De Clercq et al., 2019). A meta-analysis also confirmed the positive relationship between job satisfaction and OCB (LePine et al., 2002). Job satisfaction is an important factor that can fuel employees' ability to do beneficial extra-role activities (Bowling, 2009). Thus, we argue that the accumulation of job satisfaction might steer employees toward doing more OCB.

Social exchange theory provides insight into the relationship between self-control and VWB. Based on social exchange theory, individuals will reciprocate with attitudes and behaviors that match the expectations of their employers (Özbek et al., 2016). Because employees with high self-control tend to have better work outcomes and higher life satisfaction (Tangney et al., 2004; Hofmann et al., 2014; Dou et al., 2016), they are more likely to perceive their relationship with the organization as a fair social exchange. Therefore, they tend to increase their attachment to the organization, leading to more OCB (Cardona et al., 2004; Jena and Goswami, 2013). Another explanation for why job satisfaction is positively linked to OCB is that the positive emotion derived from job satisfaction contributes to OCB (Miles et al., 2002).

According to social exchange theory, job dissatisfaction implies a perceived imbalance in which the benefits that employees gain from the organization cannot compensate for their contribution. Employees who are dissatisfied with their jobs are more likely to display CWB because they are more inclined to perceive an imbalance in the workplace and make negative attributions (Zhang and Deng, 2016; Cohen and Diamant, 2019). Hofmann et al. (2014) reported that people with higher levels of self-control are more likely to be satisfied with their lives because they are better able to regulate negative emotions, manage conflict, and balance their daily lives. This argument leads us to suggest that employees' job satisfaction can contribute to their self-control ability. That is, employees with higher levels of self-control are better able to regulate their negative emotions from work, which might lead to higher job satisfaction, and these employees may therefore be less likely to display CWB. Another possibility is that employees who have low level of job satisfaction did not care about losing their jobs as much as those who have a high level of job satisfaction. On the other hand, satisfied employees did not risk losing their job by displaying CWB (Bowling, 2009). Finally, low level of job satisfaction may generate negative emotions that would contribute to CWB. This explanation is consistent with previous studies suggesting an emotion-centered model to explain extra-role behaviors (Spector and Fox, 2002). Based on the above considerations, the following can be hypothesized:

***Hypothesis 2****: Job satisfaction mediates the relationship between self-control and OCB*.

***Hypothesis 3****: Job satisfaction mediates the relationship between self-control and CWB*.

### The Present Study

**Figure 1:**
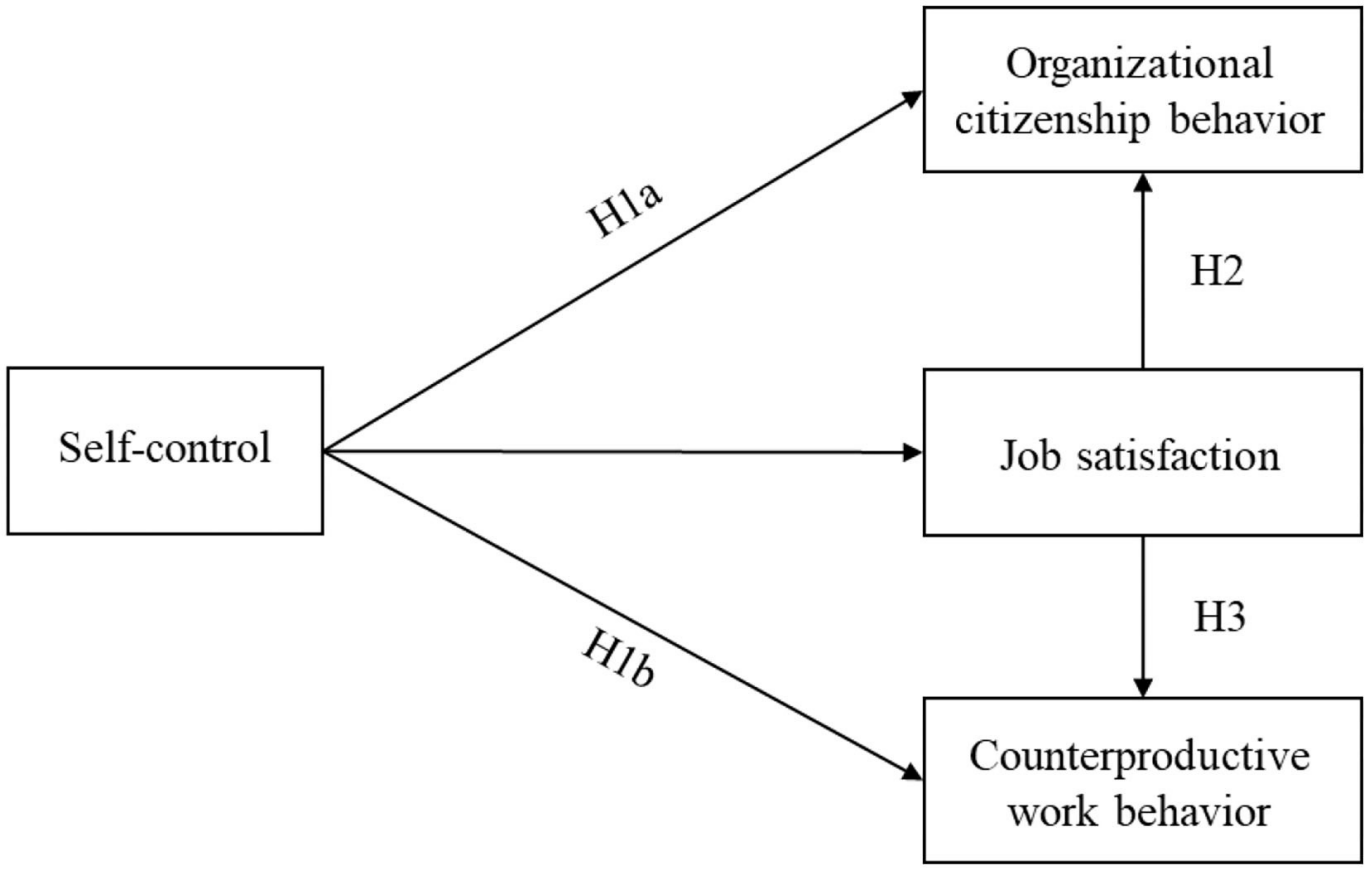
Theoretical model.

In the present study, we aimed to investigate the relationship between self-control and VBW and the mediating role of job satisfaction among Chinese employees (see in Figure 1). In line with the aforementioned literature, we expected that (1) trait self-control is positively related to OCB and negatively related to CWB; (2) job satisfaction mediates the trait self-control-OCB link; and (3) job satisfaction mediates the trait self-control–CWB link. By exploring the relationship between self-control and VWB and the possible mediating role of job satisfaction, the present study aimed to contribute to the current limited literature on the relationship between self-control and OCB/CWB and add to the understanding of the underlying mechanisms of OCB/CWB.

## Methods

### Participants and Procedures

Data were collected from a variety of organizations in southeastern China. In total, 1,167 full-time employees (536 males, 631 females) were willing to complete the surveys via an online advertisement (www.wjx.cn). After some copies of questionnaires were excluded because they contained too many identical answers, 1,101 valid surveys remained for the analysis (a response rate of 94.3%). Among these 1,101 employees (493 males, 608 females), the participants ranged in age from 18 to 64 years (*M* = 30.03 years, *SD* = 6.53 years), and the average work tenure was 4.47 years (*SD* = 5.07). These individuals provided consent prior to answering self-report measures online to enter a drawing for 100 RMB (~15 U.S. dollars).

Due to the cross-sectional nature of the design, we implemented several procedures to mitigate concerns of common method bias and social desirability bias (Podsakoff et al., 2003). According to the recommendations proposed by Podsakoff et al. (2003), which are aimed at reducing demand characteristics and evaluation apprehension, all participants were assured that their responses would be confidential, and the data they provided would be used only for research purposes. We also followed recommendations suggested by Conway and Lance (2010), which include utilizing counterbalancing of measures and demonstrating adequate evidence of measure reliability. All procedures involving human participants were reviewed and approved by the research ethics committee in the School of Education at Guangzhou University (Protocol Number: GZHU2017002).

### Measures

All English-based measures were translated into Chinese based on translation/back-translation procedures. The coefficient alpha for each scale is presented in Table 1.

**Table 1:** Descriptive statistics, correlations, and reliabilities among variables.

	***M***	***SD***	**1**	**2**	**3**	**4**	**5**	**6**	**7**
1. Age	29.96	6.67	—						
2. Gender	0.45	0.50	0.11^***^	—					
3. Monthly income	4558.26	4758.20	0.12^***^	0.03	—				
4. Self-control	3.41	0.69	0.11^***^	−0.05	0.02	(0.78)			
5. Job satisfaction	3.51	0.64	0.03	−0.01	0.06^*^	0.22^***^	(0.93)		
6. OCB	5.60	0.97	0.11^***^	−0.04	0.08^*^	0.40^***^	0.49^***^	(0.78)	
7. CWB	1.49	0.47	−0.04	−0.06^*^	0.06^*^	−0.41^**^	−0.33^***^	−0.38^***^	(0.87)

*N = 1,101. Gender was dummy-coded as 0 (=female) and 1 (=male). Cronbach's alpha are reported along the diagonal*.

* p < 0.05;

** p < 0.01;

*** *p < 0.001*.

#### Self-Control

Self-control was measured with the 8-item scale revised by Maloney et al. (2012), which from the 13-item *Brief Self-Control Scale* (BSCS) developed by Tangney et al. (2004). The items were rated on a 5-point scale from 1 (*not like me at all*) to 5 (*very much like me*). The average score for all items was calculated, and a higher score indicates better self-control. BSCS has been shown to have good psychometric properties in different cultures (Dou et al., 2016; Situ et al., 2016; Singh and Göritz, 2019). Sample items include “I am good at resisting temptation” and “I do certain things that are bad for me, if they are fun.”

#### Job Satisfaction

*Job satisfaction* was measured with a short form of the *Minnesota Job Satisfaction Questionnaire* (MSQ) developed by Arvey et al. (1989). This scale includes 20 items measured on a 5-point scale from 1 (*very dissatisfied*) to 5 (*very satisfied*). The average score for all items was calculated, and a higher score indicates greater job satisfaction. The MSQ can be applied to evaluate job satisfaction, drawing attention to key work facets and grouping them into two dimensions: intrinsic satisfaction (e.g., being able to keep busy all the time) and extrinsic satisfaction (e.g., the way my boss handles people).

#### Organizational Citizenship Behaviors

Positive voluntary behavior at work was measured with the *Organizational Citizenship Behavior Scale* (OCBS) developed by Aryee et al. (2002). This scale consists of 9 items rated on a 7-point scale from 1 (*strongly disagree*) to 7 (*strongly agree*). The average score for all items was calculated, and a higher score indicates more OCB. Sample items include “makes suggestions to improve work procedures” and “expresses opinions honestly when others think differently.”

#### Counterproductive Work Behavior

Negative voluntary behavior at work was measured with the 10-item short version of the *Counterproductive Work Behavior Checklist* (CWBC) developed by Spector et al. (2010). This measure lists a set of behaviors and asks respondents how often they have engaged in such behavior on a 5-point scale from 1 (*never*) to 5 (*every day*). A higher score indicates more negative voluntary behavior at work. Sample items include “Purposely wasted your employer's materials/supplies” and “Insulted or made fun of someone at work.”

#### Control Variables

Consistent with previous research (Ng et al., 2016), we measured *age, gender*, and *income* to control for their potentially spurious effects.

### Data Analysis

The percentage of missing data was under 2% in the valid sample, and missing data points were handled by using the full information maximum likelihood (FIML; Acock, 2005). First, descriptive statistics were performed in SPSS 22.0 to estimate the levels of participants' self-control, job satisfaction, OCB, and CWB. Second, correlation analyses were carried out to capture the associations among self-control, job satisfaction, OCB, and CWB. The correlation coefficients of 0.10, 0.30, and 0.50 represent small, medium, and large effect sizes, respectively (Cohen, 1992). Finally, the hypothesized mediation model was tested via path analysis using Mplus 7.0. We drew 5,000 bootstrapping samples and used 95% confidence intervals to determine the significance of the mediation. The mediating effect was considered significant if the 95% confidence interval excluded zero (Preacher and Hayes, 2008). Furthermore, we initially conducted confirmatory factor analyses (CFAs) to test for construct distinctiveness.

## Results

### Measurement Model and Common Method Variance

By following the recommendations of Conway and Lance (2010), we conducted a series of CFAs to examine the discriminant validity of the employees' four self-reported variables (both independent and dependent).

As shown in Table 2, the assumed four-factor model (self-control, job satisfaction, OCB, and CWB) provided a better fit to the data than any other model, including a model in which self-control and job satisfaction were combined but the two dependent variables were modeled as separate factors (M_2_), Δχ^2^ (33) = 4,120.62, *p* < 0.001, including a model in which self-control and job satisfaction were combined and the two dependent variables were combined (M_3_), Δχ^2^ (35) = 6236.28, *p* < 0.001. The four-factor model was also superior to a one-factor model that combined all four variables into one factors (M_2_), Δχ^2^ (36) = 8558.96, *p* < 0.001. We concluded that the four variables were empirically distinct from one another, representing four distinct constructs. Therefore, results did not provide evidence for common method bias.

**Table 2:** Model fit results for measurement model comparison.

**Variable**	**χ^**2**^**	***df***	**Δχ^**2**^ (Δ*df*)**	**RMSEA**	**CFI**	**NNFI**	**SRMR**
M_1_: SC; JS; OCB; CWB	3011.69	998	—	0.04	0.91	0.90	0.07
M_2_: SC + JS; OCB; CWB	7132.31	1,031	4120.62 (33)	0.07	0.72	0.71	0.09
M_3_: SC + JS; OCB + CWB	9247.97	1,033	6236.28 (35)	0.09	0.63	0.61	0.10
M_4_: SC + JS + OCB + CWB	11570.65	1,034	8558.96 (36)	0.10	0.52	0.50	0.10

*N = 1,101. SC, self-control; JS, job satisfaction; OCB, organizational citizenship behavior; CWB, counterproductive work behavior. All alternative models were compared with the hypothesized four-factor model. All Δχ^2^ are significant at p < 0.01*.

### Descriptive Statistics and Correlations

Means, standard deviations, correlations, and coefficient alphas of the variables are presented in Table 1. The results show that self-control was positively related to job satisfaction (*r* = 0.22, *p* < 0.001) and OCB (*r* = 0.40, *p* < 0.001) and negatively related to CWB(*r* = −0.41, *p* < 0.001). Job satisfaction was positively association with OCB (*r* = 0.49, *p* < 0.001) but negatively associated with CWB (*r* = −0.33, *p* < 0.001). These findings provide preliminary support for the hypothesized relationships.

### Testing the Direct and Mediational Pathways

#### Direct Effects

We first assessed the direct effect of self-control on OCB/CWB. Model results consistent with Hypotheses 1a and 1b indicated that self-control was associated with higher levels of employees' OCB (β = 0.41, *p* < 0.001) but associated with lower levels of employees' CWB (β = −0.40, *p* < 0.001) after controlling for age, gender, and monthly income.

#### Mediational Effects

**Figure 2:**
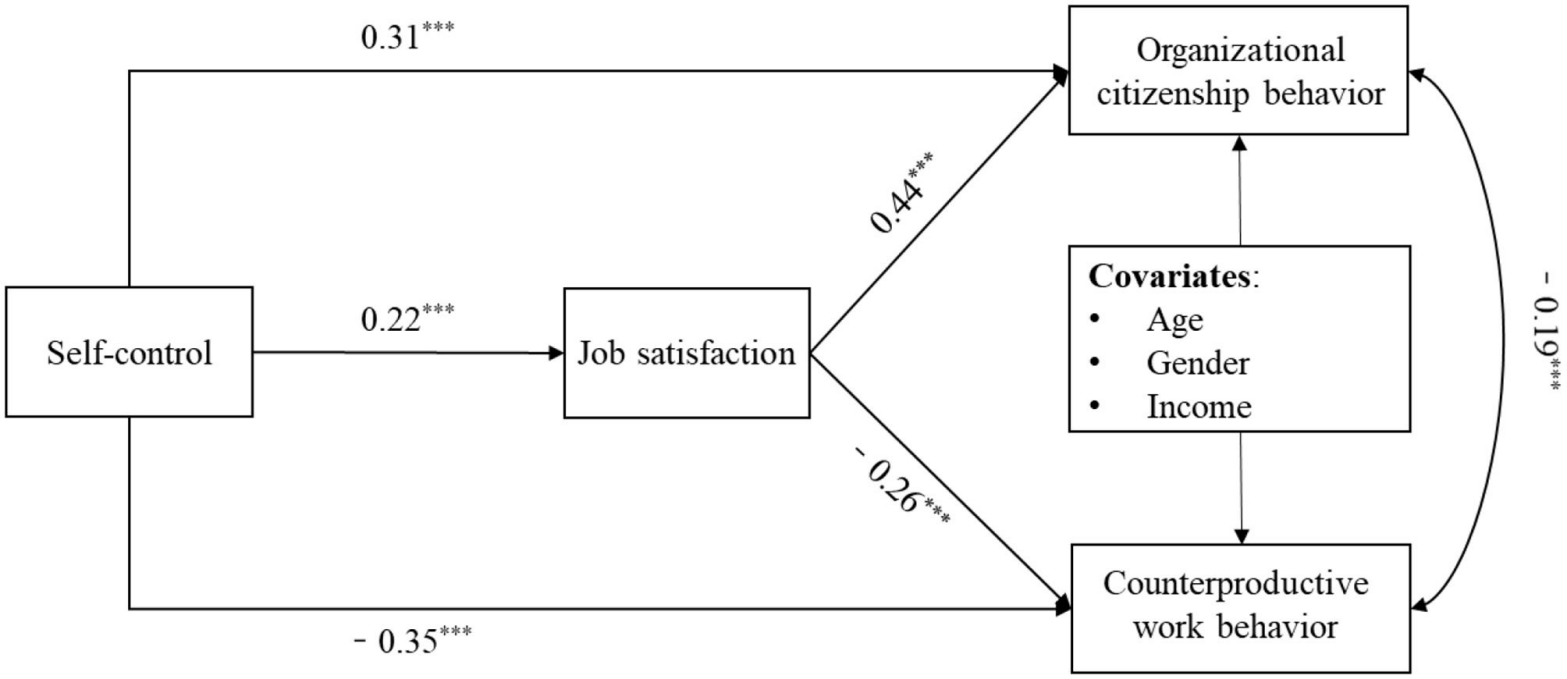
Mediation model from self-control to employees' OCB and CWB. Standardized coefficients are reported; ***p < 0.001.

Next, the mediational model was tested which included employees' job satisfaction. The model fit to the data well, χ^2^ = 3.97, *df* = 3, RMSEA = 0.017 with 90% CI = (0.001, 0.057), CFI = 0.99, SRMR = 0.011. As shown in Figure 2, employees' job satisfaction mediated the relationship between self-control on OCB and CWB after controlling for age, gender, and monthly income. That is, self-control significantly predicted employees' job satisfaction (β = 0.22, *p* < 0.001). Also, employees' job satisfaction was positively related to OCB (β = 0.44, *p* < 0.001), while it was negatively related to CWB (β = −0.22, *p* < 0.001). Finally, as shown in Table 3, the bias-corrected bootstrapping test of the indirect effects indicated that job satisfaction significantly mediated the link between self-control and OCB [estimate = 0.094, S.E. = 0.016, 95% CI = (0.063, 0.125)] also significantly mediated the link between self-control and CWB [estimate = −0.057, S.E. = 0.011, 95% CI = (−0.077, −0.036)]. Thus, Hypotheses 2 and 3 were supported.

**Table 3:** The specific indirect effect for each indirect pathway in the model based on the bias-corrected bootstrapped estimates.

**Specific pathways tested in the model**	**Bias-corrected bootstrapped estimates for the effects**			
	**Unstandardized**	**SE**	**95% CI**	**Standardized**
**Direct pathway**				
Self-control → OCB	0.545	0.041	(0.463, 0.625)	0.404
Self-control → CWB	−0.275	0.022	(−0.320, −0.234)	−0.403
**Indirect pathways**				
IND 1: self-control → job satisfaction → OCB	0.094	0.016	(0.063, 0.125)	0.137
IND 2: self-control → job satisfaction → CWB	−0.057	0.011	(−0.077, −0.036)	−0.082

*OCB, organizational citizenship behavior; CWB, counterproductive work behavior. Indirect pathways are significant based on the bias-corrected bootstrapped 95% CI. According to Kenny (2012), standardized indirect effects around 0.01 were “small,” effects around 0.09 were “medium,” and effect around 0.25 were “large”*.

## Discussion

The present research sought to reveal the relationship between self-control and VWB (OCB/CWB) and the mediating role of job satisfaction in Chinese employees. As expected, we found that self-control was positively related to OCB and negatively associated with CWB, consistent with the findings of previous studies (Bolino et al., 2012; Fehr et al., 2017; Wang et al., 2017). We further found that job satisfaction mediated the relationship between self-control and OCB/CWB. This finding reveals the internal mechanism of the effect of self-control on employees' VWB. Our results enrich and broaden the literature on employees' VWB in a collectivist cultural background from the perspective of employees' personal traits and work attitudes.

### Theoretical Implications

There were some notable theoretical implications of the present study.

First, consistent with Wang et al. (2017), we confirmed the relationship between self-control and OCB and confirmed that employees with high self-control were more likely to refrain from detrimental behavior than employees with lower levels of self-control. Therefore, the Chinese idiom “self-controlling and public-spirited” was verified from the positive and negative aspects; that is, compared with employees with lower levels of self-control, employees with better self-control ability contribute more to the organization by not only demonstrating more OCB but also refraining from CWB.

Second, the present study contributes to the growing literature on trait self-control and VWB. The present research found that in addition to external approaches, such as organizational climate and leadership style [e.g., Wu et al. (2016) and Newman et al. (2017)], personal traits such as self-control can enable employees to engage in OCB or refrain from CWB. As personal factors, self-control provides a novel way to understanding employees' behavior in the work setting.

Another theoretical contribution of the present study is that we revealed the mediating role of job satisfaction, which helps to better understand the mechanism underlying the relationship between self-control and VWB. This finding supports social exchange theory, suggesting that compared with employees with low self-control, employees with better self-control ability tend to have a higher satisfaction with their work, which in turn motivates employees to engage in more OCB (Bolino et al., 2012). In contrast, if employees do not feel that they are gaining the expected benefits from their organization, they tend to engage in more CWB (Cardona et al., 2004).

### Practical Implications

The current research has several implications for human resource management practices:

First, the study highlights the important effect of employees' personal traits (self-control) on their behavior in the organization. Based on this finding, organizations should pay more attention to employees' self-control ability. For example, in the process of employee recruitment, organizations can take interviewees' self-control ability into account by applying self-control tests. Second, when selecting candidates for promotions, important positions, or assignments, organizations can consider employees' self-control ability as a predictor of greater OCB. Additionally, organizations can provide specific training to improve employees' self-control ability, which can benefit both the organization and employees in the long term.

Previous studies have shown that motivation can have an impact on the implementation of self-control (Baay et al., 2014). From this perspective, an organization can motivate employees to implement their self-control ability by using certain strategies. For example, organizations can guide employees to combine their career planning with organizational development. By developing greater attachment to the organization, employees will feel more motivated, leading to an increase in the application of self-control.

### Limitations and Future Directions

Several limitations of the current study should be noted. First, because the present study was cross-sectional, the causal relationship between self-control and VWB could not be revealed. Thus, future studies could adopt a longitudinal design. A second limitation concerns the self-reported nature of the study. Although every effort was made to avoid the problem of common method variance by assuring anonymity and employing unified instructions, we still cannot exclude the potential issues with employees' self-reported data. Therefore, to improve the ecological validity of the research, future study is needed to further extend and optimize the measurement (e.g., by self-reports, peer assessments, and behavioral experiments). Third, the literature on the mediating mechanism in the relationship between self-control and VWB is limited. Future studies may also examine other possible mediators to better understand VWB. Finally, given the practical implications of our findings, several practical issues require further attention. Although we revealed that self-control plays an important role in OCB, research on methods for self-control inventions is needed to better apply self-control for practical use.

## Data Availability Statement

The datasets generated for this study are available on request to the corresponding author.

## Ethics Statement

The studies involving human participants were reviewed and approved by Ethics Review Committee of Education School, Guangzhou University. The patients/participants provided their written informed consent to participate in this study.

## Author Contributions

Y-JW and KD: conceptualization, data curation, and investigation. Y-JW, K-YC, and KD: formal analysis, methodology, software, and writing—original draft. KD: project administration. Y-JW, KD, and Y-ZL: resources. KD and Y-ZL: supervision. Y-JW, K-YC, KD, and Y-ZL: writing—review and editing. All authors contributed to the article and approved the submitted version.

## Conflict of Interest

The authors declare that the research was conducted in the absence of any commercial or financial relationships that could be construed as a potential conflict of interest.
