# Supplementary material for: Linking Self-Control to Voluntary Behaviors at Workplace: The Mediating Role of Job Satisfaction
Source: Front Psychol. 2021 Mar 23;12:530297. doi: 10.3389/fpsyg.2021.530297 (PMC8021765; doi:10.3389/fpsyg.2021.530297)
Supplement: Supplementary file 1 [file Table_1.DOCX]

**Appendix**

**(1) Brief Self-Control Scale**

| **Instructions:** Using the scale provided, please indicate how much each of the following statements reflects how you typically are. | |
| --- | --- |
|  | **Not at all Very much** |
| 1. I am good at resisting temptation.  我善于抵制诱惑 | 1**———**2**———**3**———**4**———**5 |
| 2. I have a hard time breaking bad habits.  我很难戒掉坏习惯 | 1**———**2**———**3**———**4**———**5 |
| 3. I do certain things that are bad for me, if they are fun.  如果有些事情很有趣，即使对我不好，我还是会做 | 1**———**2**———**3**———**4**———**5 |
| 4. I wish I had more self-discipline.  我不够自律 | 1**———**2**———**3**———**4**———**5 |
| 5. People would say that I have iron self- discipline.  其他人认为我是一个意志坚定的人 | 1**———**2**———**3**———**4**———**5 |
| 6. Pleasure and fun sometimes keep me from getting work done.  我有时会因为沉迷一些愉快和有趣的事情而无法按时完成任务 | 1**———**2**———**3**———**4**———**5 |
| 7. Sometimes I can’t stop myself from doing something, even if I  know it is wrong.  有时，即使我知道一些事情是错的，我也忍不住去做 | 1**———**2**———**3**———**4**———**5 |
| 8. I often act without thinking through all the alternatives.  我经常没有考虑周全就行动 | 1**———**2**———**3**———**4**———**5 |

**(2) Minnesota Job Satisfaction Questionnaire**

| **Ask yourself:** How **satisfied** am I with this aspect of my job? | |
| --- | --- |
|  | **Very dissatisfied Very satisfied** |
| 1. Being able to keep busy all the time.  能够一直保持忙碌的状态 | 1**———**2**———**3**———**4**———**5 |
| 2. The chance to work alone on the job.  独立工作的机会 | 1**———**2**———**3**———**4**———**5 |
| 3. The chance to do different things from time to time.  时不时地能有做一些不同事情的机会 | 1**———**2**———**3**———**4**———**5 |
| 4. The chance to be somebody in the community.  在团体中成为重要角色的机会 | 1**———**2**———**3**———**4**———**5 |
| 5. The way my boss handles people.  我的老板对待他/她的下属的方式 | 1**———**2**———**3**———**4**———**5 |
| 6. Competence of my supervisor in making decisions.  我的上司做决策的能力 | 1**———**2**———**3**———**4**———**5 |
| 7. Being able to do things that don’t go against my conscience.  能够做一些不违背我良心的事情 | 1**———**2**———**3**———**4**———**5 |
| 8. The way my job provides for steady employment.  我的工作稳定性 | 1**———**2**———**3**———**4**———**5 |
| 9. The chance to do things for others.  能够为其他人做些事情的机会 | 1**———**2**———**3**———**4**———**5 |
| 10. The chance to tell people what to do.  告诉他人该做些什么的机会 | 1**———**2**———**3**———**4**———**5 |
| 11. The chance to do something that makes use of my abilities.  能够充分发挥我能力的机会 | 1**———**2**———**3**———**4**———**5 |
| 12. The way company policies are put into practice.  公司政策实施的方式 | 1**———**2**———**3**———**4**———**5 |
| 13. My pay and the amount of work I do.  我的收入与我的工作量 | 1**———**2**———**3**———**4**———**5 |
| 14. The chances for advancement on this job.  职位晋升的机会 | 1**———**2**———**3**———**4**———**5 |
| 15. The freedom to use my own judgment.  能自己作出判断的自由 | 1**———**2**———**3**———**4**———**5 |
| 16. The chance to try my own methods of doing the job.  自主决定如何完成工作的机会 | 1**———**2**———**3**———**4**———**5 |
| 17. The working conditions.  工作条件 | 1**———**2**———**3**———**4**———**5 |
| 18. The way coworkers get along with each other.  同事之间相处的方式 | 1**———**2**———**3**———**4**———**5 |
| 19. The feeling of accomplishment I get from the job.  工作表现出色时，所获得的奖励 | 1**———**2**———**3**———**4**———**5 |
| 20. The feeling of accomplishment I get from the job.  我能够从工作中获得的成就感 | 1**———**2**———**3**———**4**———**5 |

**(3) Organizational Citizenship Behavior Scale**

| **Instructions:** Please use a 7-point scale (1=strongly disagree, 7=strongly agree) how likely you are to perform each of these behaviors. | |
| --- | --- |
|  | **Strongly disagree Strongly agree** |
| 1. Helped new employees get oriented to the job.  我乐于帮助新同事尽快适应工作环境 | 1**——**2**——**3**——**4**——**5**——6——7** |
| 2. Willingly give your time to help others who have work-related problems.  我总愿意帮助同事解决工作中的问题 | 1**——**2**——**3**——**4**——**5**——6——7** |
| 3. Helped a co-worker who had too much to do.  若需要，我愿意为同事分担部分工作 | 1**——**2**——**3**——**4**——**5**——6——7** |
| 4. Helped co-worker learn new skills or shared job knowledge.  平时我很乐意与同事沟通协调 | 1**——**2**——**3**——**4**——**5**——6——7** |
| 5. Makes suggestions to improve work procedures  即使没人看到，我也总是自觉遵守规章制度 | 1**——**2**——**3**——**4**——**5**——6——7** |
| 6. Take my work seriously and rarely make mistakes.  我总是认真对待工作，极少犯错 | 1**——**2**——**3**——**4**——**5**——6——7** |
| 7. I willingly to take on challenging work.  我不介意新的或很有挑战性的工作 | 1**——**2**——**3**——**4**——**5**——6——7** |
| 8. Make innovative suggestions to improve department.  为提高工作效率和质量，我坚持学习 | 1**——**2**——**3**——**4**——**5**——6——7** |
| 9. Came in early or stayed late without pay to complete a project or task.  我经常早早上班，并立即投入工作 | 1**——**2**——**3**——**4**——**5**——6——7** |

**(4) Counterproductive Work Behavior Checklist**

| **Instructions:** How often have you done each of the following things on your present job? | |
| --- | --- |
|  | **Never Every day** |
| 1. Purposely wasted your employer’s materials/supplies.  故意浪费单位的物资(如打印纸等) | 1**———**2**———**3**———**4**———**5 |
| 2. Complained about insignificant things at work.  抱怨在工作中做一些无关重要的工作 | 1**———**2**———**3**———**4**———**5 |
| 3. Told people outside the job what a lousy place you work for.  跟别人说自己的公司很差劲 | 1**———**2**———**3**———**4**———**5 |
| 4. Came to work late without permission.  没经批准上班迟到 | 1**———**2**———**3**———**4**———**5 |
| 5. Stayed home from work and said you were sick when you weren’t.  假装生病不去上班 | 1**———**2**———**3**———**4**———**5 |
| 6. Insulted someone about their job performance.  侮辱某些同事的工作表现 | 1**———**2**———**3**———**4**———**5 |
| 7. Made fun of someone’s personal life.  取笑某些同事的个人生活 | 1**———**2**———**3**———**4**———**5 |
| 8. Ignored someone at work.  在工作中忽略某些同事的作用 | 1**———**2**———**3**———**4**———**5 |
| 9. Started an argument with someone at work.  在工作中和某些同事争吵 | 1**———**2**———**3**———**4**———**5 |
| 10. Insulted or made fun of someone at work.  在工作中侮辱或取笑某些同事 | 1**———**2**———**3**———**4**———**5 |
